# Continued versus discontinued oxytocin stimulation in the active phase of labour (CONDISOX): individual management based on artificial intelligence - a secondary analysis

**DOI:** 10.1186/s12884-024-06461-8

**Published:** 2024-04-19

**Authors:** Sidsel Boie, Julie Glavind, Pinar Bor, Philip Steer, Anders Hammerich Riis, Bo Thiesson, Niels Uldbjerg

**Affiliations:** 1 Department of Obstetrics and Gynaecology, Randers Regional Hospital, Randers, Denmark; 2 Department of Obstetrics and Gynaecology, Aarhus University Hospital, Aarhus, Denmark; 3 Academic Department of Obstetrics and Gynaecology, Chelsea and Westminster Hospital, Imperial College London, London, UK; 4 Enversion A/S, Aarhus, Denmark; 5 Department of Clinical Medicine, Aarhus University, Aarhus, Denmark

**Keywords:** Oxytocin, Induction of labour, Mode of delivery, Prediction model, Artificial intelligence, Explainable AI

## Abstract

**Background:**

Current guidelines regarding oxytocin stimulation are not tailored to individuals as they are based on randomised controlled trials. The objective of the study was to develop an artificial intelligence (AI) model for individual prediction of the risk of caesarean delivery (CD) in women with a cervical dilatation of 6 cm after oxytocin stimulation for induced labour. The model included not only variables known when labour induction was initiated but also variables describing the course of the labour induction.

**Methods:**

Secondary analysis of data from the CONDISOX randomised controlled trial of discontinued vs. continued oxytocin infusion in the active phase of induced labour. Extreme gradient boosting (XGBoost) software was used to build the prediction model. To explain the impact of the predictors, we calculated Shapley additive explanation (SHAP) values and present a summary SHAP plot. A force plot was used to explain specifics about an individual’s predictors that result in a change of the individual’s risk output value from the population-based risk.

**Results:**

Among 1060 included women, 160 (15.1%) were delivered by CD. The XGBoost model found women who delivered vaginally were more likely to be parous, taller, to have a lower estimated birth weight, and to be stimulated with a lower amount of oxytocin. In 108 women (10% of 1060) the model favoured either continuation or discontinuation of oxytocin. For the remaining 90% of the women, the model found that continuation or discontinuation of oxytocin stimulation affected the risk difference of CD by less than 5% points.

**Conclusion:**

In women undergoing labour induction, this AI model based on a secondary analysis of data from the CONDISOX trial may help predict the risk of CD and assist the mother and clinician in individual tailored management of oxytocin stimulation after reaching 6 cm of cervical dilation.

## Introduction

When the active phase of labour has been established in women undergoing labour induction with oxytocin, discontinuation of the oxytocin reduces the risk of uterine hyperstimulation and the risk of abnormal fetal heart rate patterns [1], although possibly at the expense of an increase in caesarean delivery rate (RR 1.17, 95% CI 0.90 to 1.53) [2]. Accordingly, it remains a matter of debate whether this increase contraindicates oxytocin discontinuation as a routine [3]. Some women and their babies may benefit from oxytocin continuation, and some may benefit from oxytocin discontinuation. Therefore, it would be preferable to refine the ‘one size fits all’ approach based on randomised controlled trials by an individual evaluation of the caesarean delivery risk for both oxytocin discontinuation and oxytocin continuation. Such an individual approach may assist the parturient and her clinical advisors in a tailored decision on discontinuation or continuation of oxytocin stimulation.

The use of artificial intelligence (AI) in obstetrics is rising [4], but only a few studies have evaluated the risk of caesarean delivery. Current models of prediction of intrapartum caesarean delivery include only variables known before the initiation of the labour induction, such as parity, gestational age, maternal BMI, Bishop score, and maternal height [5–7] but not intrapartum variables. The AI approach benefits from the ability to detect complex interactions between predictors that are difficult to model with ordinary statistics [8–10].

We used AI modelling to predict the risk of caesarean delivery with continued or discontinued oxytocin stimulation in the active phase of labour using both variables known when labour induction was initiated (e.g. parity, gestational age, maternal height, maternal BMI, and estimated birthweight), and variables obtained during the labour induction (e.g. pyrexia, volume of oxytocin, total dose of oral prostaglandins given, and epidural use). We hypothesized that we could improve predictive ability by including variables reflecting the course of labour such as the dose of oxytocin used to stimulated contraction or pyrexia during labour.

## Methods

### Study population

**Fig. 1:**
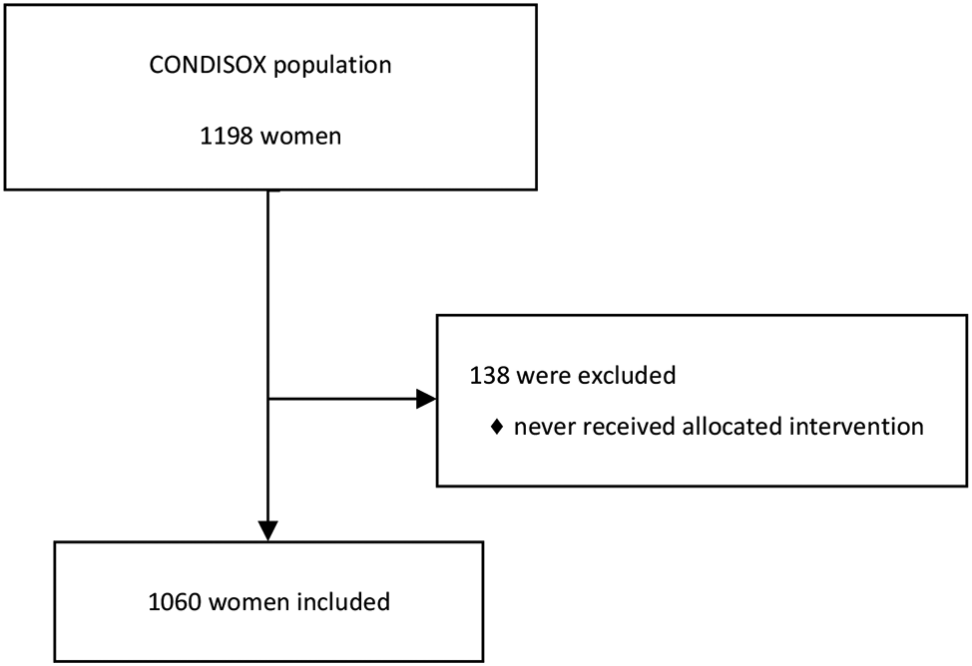
Flowchart studypopulation

The study cohort was 1198 women included in the CONDISOX trial, which was a double-blinded, placebo controlled randomised trial conducted at ten birth sites in Denmark and The Netherlands between April 2016 and June 2020 [2, 11]. The women had a term singleton pregnancy with a fetus in cephalic presentation and were stimulated with oxytocin as part of the procedure for induction of labour, before they were randomised in the active phase of labour to either continued or discontinued stimulation in a double-blind placebo-controlled trial. For this secondary study, we excluded 138 randomised women who never received the allocated intervention due to very rapid progress of labour, see Fig. 1. The Danish obstetrical practice differs from many other parts of the world by having universal prenatal care, free of charge, and outpatient induction regimes in low-risk pregnancies, often including oral misoprostol or a cervical ripening catheter. Further details regarding the labour and induction protocol can be found in the original paper and the published trial protocol [2, 11].

### Predictors

The CONDISOX trial data had detailed demographic and clinical information available for evaluation. We included the clinical characteristics of both the mother, the pregnancy, the fetus (when still unborn) and labour (until 6 cm cervical dilatation) as predictors (Table 1). All data were collected prospectively and entered either directly into the study database or into the electronic patient medical file and amalgamated in the study database no later than 30 days postpartum. Data on maximum oxytocin dose (IU/min) was not collected in the initial part of the original trial.

**Table 1:** Maternal and labour characteristics for women giving birth vaginally or by CD

Characteristic	Vaginal *N* = 900 (84.9%)	Caesarean *N* = 160 (15.1%)	Total *N* = 1060 (100%)
Allocated treatment in active phase of labour			
Placebo	449 (49.9)	86 (53.8)	535 (50.5)
Oxytocin	451 (50.1)	74 (46.2)	525 (49.5)
Parity			
0	596 (66.2)	136 (85.0)	732 (69.1)
1	192 (21.3)	18 (11.2)	210 (19.8)
>1	112 (12.4)	6 (3.8)	118 (11.1)
Indication for oxytocin			
Induced labour*	561 (62.3)	114 (71.2)	675 (63.7)
Prelabour rupture of membranes	339 (37.7)	46 (28.7)	385 (36.3)
Maternal pyrexia during labour**			
No	821 (91.2)	124 (77.5)	945 (89.2)
Yes	79 (8.8)	35 (21.9)	114 (10.8)
Previous CD			
No	833 (92.6)	144 (90.0)	977 (92.2)
Yes	67 (7.4)	16 (10.0)	83 (7.8)
Missing	0 (0.0)	1 (0.6)	1 (0.1)
Maternal height (cm)			
Mean (SD)	170.3 (39.7)	165.7 (6.8)	169.6 (36.7)
Maternal pre-pregnancy weight (kg)			
Mean (SD)	76.7 (47.0)	74.6 (17.2)	76.3 (43.8)
Maternal pre-pregnancy BMI (kg/m^2^)			
Mean (SD)	28.4 (46.2)	27.1 (5.9)	28.2 (42.6)
Smoking during pregnancy			
Missing	18 (2.0)	2 (1.2)	20 (1.9)
No	742 (82.4)	141 (88.1)	883 (83.3)
Yes	140 (15.6)	17 (10.6)	157 (14.8)
Marital status			
co-habiting	474 (52.7)	96 (60.0)	570 (53.8)
married	334 (37.1)	49 (30.6)	383 (36.1)
single	80 (8.9)	13 (8.1)	93 (8.8)
Missing N (%)	12 (1.3)	2 (1.2)	14 (1.3)
Estimated birthweight (g)			
Mean (SD)	3548 (395.6)	3724 (352.7)	3575 (394.4)
Missing N (%)	72 (8)	10 (6)	978 (92)
Gestational diabetes			
No	820 (91.1)	140 (87.5)	960 (90.6)
Yes	80 (8.9)	20 (12.5)	100 (9.4)
Hypertension during pregnancy***			
No	813 (90.3)	149 (93.1)	962 (90.8)
Yes	87 (9.7)	11 (6.9)	98 (9.2)
Preeclampsia			
No	846 (94.0)	144 (90.0)	990 (93.4)
Yes	54 (6.0)	16 (10.0)	70 (6.6)
Inflammatory bowel disease			
No	869 (96.6)	153 (95.6)	1,022 (96.4)
Yes	31 (3.4)	7 (4.4)	38 (3.6)
Small for gestational age^21^			
No	878 (97.6)	159 (99.4)	1,037 (97.8)
Yes	22 (2.4)	1 (0.6)	23 (2.2)
Autoimmune disease ****			
No	883 (98.1)	157 (98.1)	1,040 (98.1)
Yes	17 (1.9)	3 (1.9)	20 (1.9)
Use of anti-depressive medication at term			
No	879 (97.7)	152 (95.0)	1,031 (97.3)
Yes	21 (2.3)	8 (5.0)	29 (2.7)
Length of gestation at birth (days)			
Mean (SD)	281.0 (9.7)	284.3 (8.9)	281.5 (9.7)
Cervical ripening: oral prostaglandins			
No	546 (60.7)	70 (43.8)	616 (58.1)
Yes	353 (39.2)	90 (56.2)	443 (41.8)
Missing	1 (0.1)	0 (0.0)	1 (0.1)
Total dose of oral prostaglandins given (µg)			
Mean (SD)	201.6 (141.6)	220.0 (147.7)	205.3 (142.9)
Missing N (%)	41 (12)	10 (11)	
Cervical ripening: cervical ripening catheter			
No	782 (86.9)	135 (84.4)	917 (86.5)
Yes	116 (12.9)	25 (15.6)	141 (13.3)
Missing	2 (0.2)	0 (0.0)	2 (0.2)
Cervical dilatation at oxytocin stimulation (cm)			
Mean (SD)	2.5 (1.0)	2.6 (1.0)	2.6 (1.0)
Missing N (%)	14 (2)	0 (0)	14 (1)
Cervical dilatation at randomisation (cm)			
Mean (SD)	7.2 (1.4)	6.6 (1.0)	7.1 (1.4)
Missing N (%)	1 (0.1)	0 (0)	1 (0.09)
Epidural use			
No	369 (41.0)	19 (11.9)	388 (36.6)
Yes	531 (59.0)	141 (88.1)	672 (63.4)
Volume of oxytocin (IU) *****			
Median (IQR)	1.7 (0.80–3.5)	2.8 (1.4–4.4)	1.8 (0.9–3.7)
Missing N (%)	81 (2)	21 (13)	102 (10)
Maximum dose of oxytocin (mIU/min) *****			
Mean (SD)	13.4 (6.7)	15.8 (7.35)	13.8 (6.9)
Missing N (%)	566 (63)	96 (60)	662 (62)
Fetal sex			
Girl	441 (49.0)	54 (33.8)	495 (46.7)
Boy	459 (51.0)	106 (66.2)	565 (53.3)

*Postdate pregnancy, hypertensive disorders, BMI ≥ 35, oligohydramnios, diabetes, maternal request etc** defined as ≥ 38.2 °C with epidural, without epidural: ≥38 °C ***repeated measures of blood pressure > 140/>90 during pregnancy **** systemic lupus erythematosus, hyperthyroidism, and hypothyroidism. ***** before the active phase of labour was reached and allocated treatment was initiated *****

### Population based risk

Extreme gradient boosting (XGBoost) [12] was used to build the model for predicting delivery by emergency caesarean delivery. XGBoost is a gradient boosting algorithm that sequentially builds a prediction model as an ensemble of several decision trees, with each new tree trained to minimize the risk residuals for the previous state of the ensemble model.

Decision trees do not require pre-processing of their input data (e.g., normalization) and therefore no scaling of data is needed before adding interaction effects. Some implementations can also handle intelligent imputations for missing values in the data. Moreover, the calculation of Shapley additive explanations (SHAP) is optimized for ensembles of decision trees like XGBoosted tree ensembles [13]. Therefore, we chose to base the prediction models on XGBoost as it handles both interactions and missing values for our data input and has optimized integration into the explanations for individual predictions from a trained model.

All predictions and associated SHAP explanations are based on models that are trained and evaluated in a five-fold cross-validation setup. We use the Area Under the Receiver Operating Characteristic (AUROC) curve and the Area Under the Precision-Recall Curve (AUPRC) to evaluate the overall predictive performance of the models[14], and we report the means and 95% confidence intervals (CI) based on the five cross-validation folds. A calibration plot (reliability curve) was used to illustrate how well predicted caesarean delivery rates calibrate with the actual rate and, therefore, the degree to which the reported prediction rates can be interpreted with confidence.

Microsoft SQL Server Management Studio version 17.5 was used for data extraction. Python 3.9.7, the package Scikit-learn version 1.0.2 was used for predictions, and SHAP package version 0.40.0 was used for explanations.

To explain the impact of the predictors, including interactions between predictors, on the likelihood of delivery by caesarean delivery, we calculated SHAP values.[15, 16]. A SHAP value is the average marginal contribution of a predictor’s value across all the possible combinations of the observed predictors, and has the property that the SHAP values for all predictors sum to the difference between the predicted output for a given individual and the expected output of the model across the population. A positive SHAP value indicates a positive effect on the prediction of delivery by caesarean delivery, and the other way around for a negative SHAP value. In other words, each SHAP value expresses the marginal effect that the observed parameter for the individual has on the final prediction as opposed to just predicting the prevalence.

The SHAP explanations are visualized in various types of plots. We used the global parameter importance (GPI) plot to explain model predictions across the entire population, where the size of a bar in the plot depicts the mean of the absolute SHAP values for a predictor across all individuals in the population. Furthermore, we used the SHAP summary plot to identify predictors with outlying values of high importance, as it shows a color-coded distribution of the SHAP values for each predictor, as opposed to the point estimate of importance in the GPI plot. The predictors are ranked by importance according to the GPI plot. All features are reviewed, and the top 20 are plotted.

The SHAP summary plot is more complicated than the GPI plot by offering additional detail about what is driving the predictor importance. The plot is comprised of dots, where each dot represents the SHAP value for an individual’s observed predictor. The vertical location of the dot defines the predictor, and the horizontal position is the computed SHAP value for that predictor. For a continuous predictor (e.g. height) the colour shows the predictors value—low values are marked in blue, high values are marked in red, values in the middle in purple, and predictors with a missing value are marked by a grey dot. If a “swarm” of dots is centred around zero, the parameter has no effect on the model output. A dichotomous predictor (e.g., nulliparity) is illustrated in the SHAP value plot with a high feature value (red-yes) or a low feature value.

### Individually based risk

A force plot is used to explain the difference in prediction that a given individual’s observed predictor values “forces” upon the model’s decision in comparison to a base value given by the prevalence in the population. The plot shows the direction and magnitude of this effect for each predictor. The contribution of each predictor to the individual’s predicted value can thereby be explored. Variables marked in red are those that increase the risk of outcome, and variables marked in blue are those that decrease the risk.

For each participant, we calculated the risk of caesarean delivery twice, first with continued oxytocin stimulation then with discontinued oxytocin stimulation, since this predictor was the only one that could be modified during labour. We agreed that a clinically relevant absolute risk difference in caesarean delivery was 5% or more.

## Results

This secondary analysis of a double-blinded randomised controlled study was based on 1060 labouring women stimulated with oxytocin for labour induction. A total of 160 women (15.1%) delivered by caesarean delivery (16.1% among women randomised to discontinued treatment and 14.1% among women randomised to continued treatment, RR 1.14, 95%CI 0.86–1.52)). Maternal and labour characteristics for the included women are presented in Table 1.

**Fig. 2:**
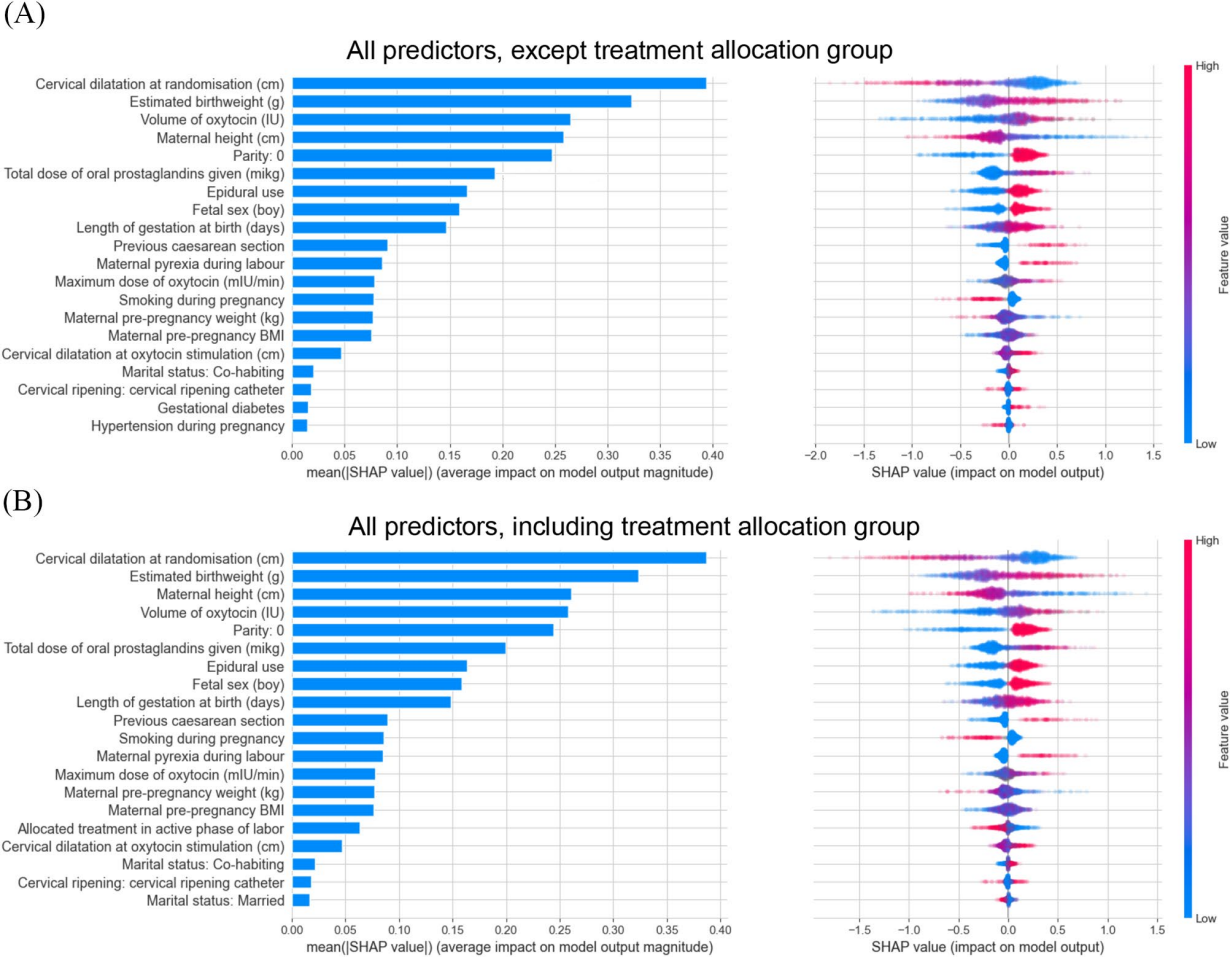
A+B SHAP summary plot Left part: SHAP summary plot, ranking all predictors of the population-based risk of CD both including (**B**) and excluding (**A**) the treatment allocation group as a predictor. Right part: SHAP summary plot, additionally offering a color-coded distribution of the SHAP values for each predictor. The top twenty predictors are listed in each plot

SHAP explanations for the XGBoost model allowed us to rank the predictors of the population-based risk of caesarean delivery (CD). The top twenty predictors are given in Fig. 2. At the very top of this ranking were predictors associated with conditions known prior to labour induction (estimated birthweight, maternal height, and parity), followed by two predictors associated with the course of labour (e.g., cervical dilatation, dose of prostaglandins and dose of oxytocin), whereas the allocation to either continuation or discontinuation of oxytocin had a relatively small but still significant mean SHAP value.

These results allowed us to deduce an algorithm for a calculation of the individually based risk of CD.

**Fig. 3:**
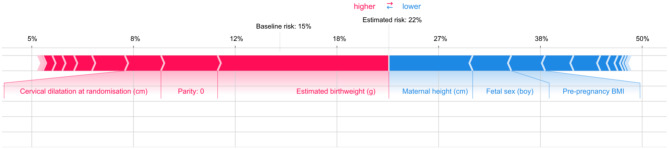
Force plot with calculation of individual based risk for one woman. Important variables for the risk increase or decrease are colored red and blue. The figure shows a woman with an estimated risk of CD of 22%. Important predictors for this relatively high risk were the estimated birthweight of 4200 g and nulliparity. Important predictors lowering the risk for this woman were the height (172 cm), the gender of the fetus (male), and the BMI of 28 kg/m^2^

**Fig. 4:**
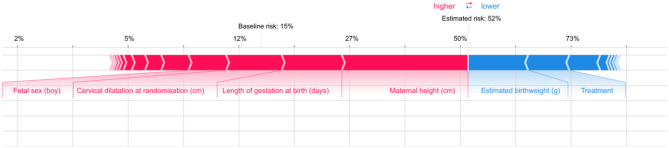
Force plot with calculation of individual based risk for one woman. Important variables for the risk increase or decrease are colored red and blue. The figure shows a woman with an estimated a risk of CD of 52%. Her risk was increased primarily by her height (148 cm) and her gestational age (287 days), whereas it was decreased by the estimated birthweight (3000 g)

- Figure 3 shows a woman with an estimated risk of CD of 22%. Important predictors for this relatively high risk were the estimated fetal weight of 4200 g and nulliparity. Important predictors lowering the risk for this woman were the height (172 cm), the gender of the fetus, and the BMI of 28 kg/m^2^.

- Figure 4 illustrates a woman with an estimated a risk of CD of 52%. Her risk was increased primarily by her height (148 cm) and her gestational age (287 days), whereas it was decreased by the estimated fetal weight (3000 g).

**Fig. 5:**
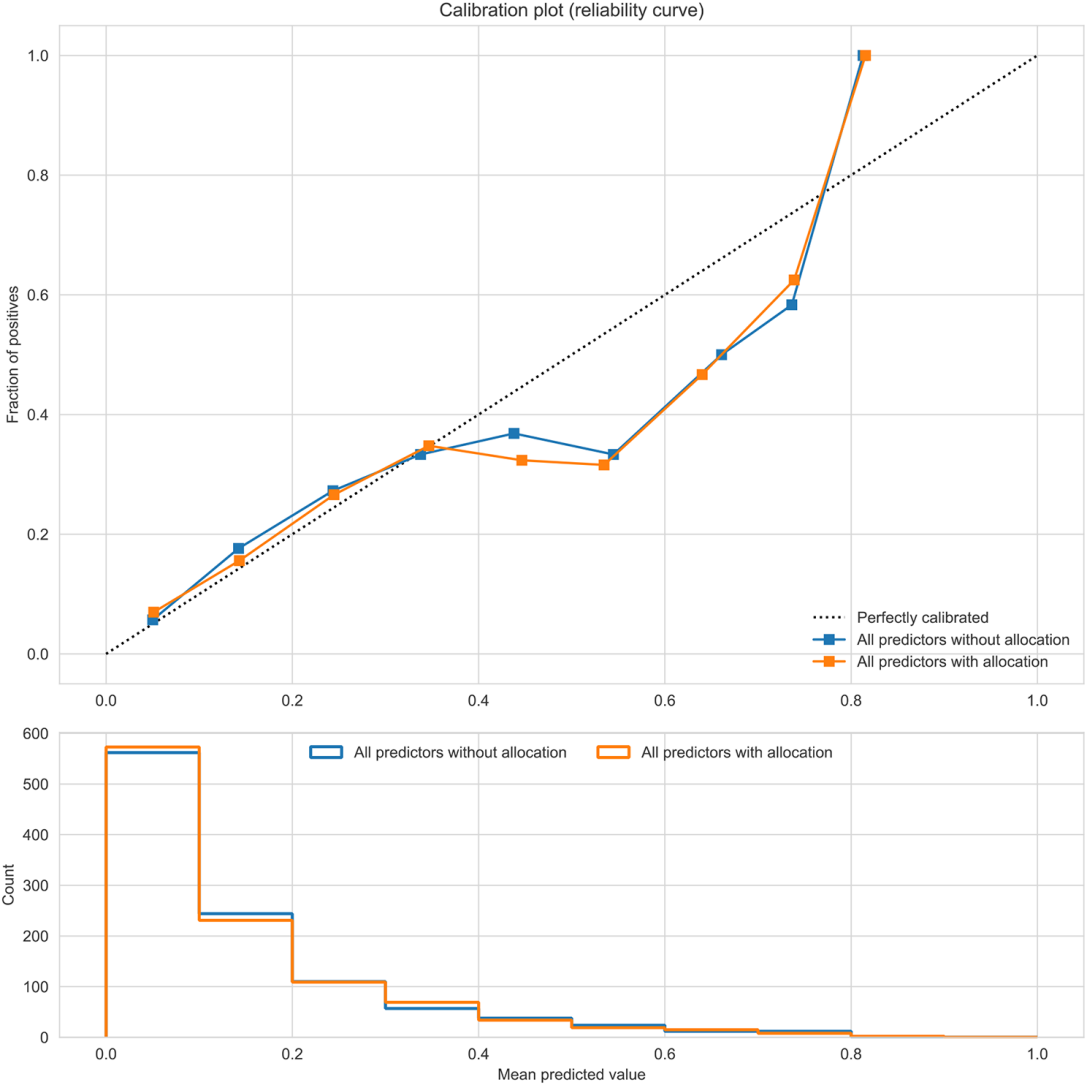
Reliability curve. Visual agreement between predicted risk of delivery by CD and women. The blue line presents all predictors excluding the treatment allocation group, whereas the orange line presents all predictors excluding the treatment allocation group

The model for delivery by CD given all predictors (except the allocated treatment) had an AUROC of 0.75 (CI: 0.71–0.79) and an AUPRC of 0.39 (CI: 0.33–0.45). Our algorithm categorized 53% of the population as low risk of CD (562 women with a risk of CD below 10%) and among these 6% ended up with a caesarean delivery. Among the 1% (13 women of 1060) with a risk above 70%, 62% (8 women of 13) had a CD (Fig. 5).

For each participant, we calculated the risk of CD twice, first with continued oxytocin stimulation then with discontinued oxytocin stimulation, since this predictor was the only one that could be modified at this stage of labour. Using a risk difference of 5% points between these two calculations as a clinically relevant cutoff, 81 women (7.6% of 1060) would benefit from oxytocin continuation whereas 27 (2.5% of 1060) would benefit from oxytocin discontinuation. Using 3% points as a clinically relevant cutoff, the model found 12.3% (130/1060) of the women in favour of continuation and 3.4% (36/1060) of the women in favour of discontinuation.

## Discussion

In this study based on women with a cervical dilatation of 6 cm during oxytocin stimulation for induced labour, we developed an algorithm to identify women with a low risk of CD as well as women with a very high risk of CD. The algorithm identified women within this population who would potentially benefit by oxytocin continuation and women who would potentially benefit from oxytocin discontinuation. For the majority of the women within this population the difference between oxytocin continuation and discontinuation in relation to the risk of CD was not clinically relevant.

It is a strength of the study that the data was collected prospectively, and that the data collected for the original trial was audited according to Good Clinical Practice (GCP). Furthermore, the proportion of missing outcomes was low. It is a weakness of the study that it is based on a secondary analysis of a randomised controlled trial. Therefore, the results need to be validated in a prospective study which could include additional variables such as the rate of progress in labour, uterine hyperstimulation, and abnormal fetal heart rate pattern. We excluded 138 women (10%) who never received the allocated treatment, primarily due to very rapid progress of labour. This could affect the internal validity of our analysis as it is likely that most women in this group would benefit from oxytocin discontinuation. Also, the external validity needs to be addressed in other populations and other settings, since our analysis was performed in women who actual reaches the active phase of labour (with a mean height of 169.6 cm and mean pre-pregnancy BMI of 28.2 kg/m^2^) and in settings where monitoring of the fetal condition and the uterine contractions were guaranteed by highly trained staff.

We found that women who delivered vaginally were more likely to be parous, induced due to PROM, they were taller, and were more likely to carry a fetus with a lower estimated birth weight. This is consistent our previous follow-on paper using this data set [17]. Two other studies have also used XGBoost for assessment of important predictors for identification of women at risk of delivery by caesarean delivery. Even though their populations differed from ours and they failed to include variables representing the course of labour, some of their findings are in line with our findings in Fig. 2. The first study evaluated women with trial of labour after caesarean (TOLAC), and the important predictors of relevance to repeat caesarean delivery included no previous vaginal delivery, low maternal height, low cervical dilatation at admission to the labour ward, and labour induction [18]. The second study evaluated 15 antepartum variables in nulliparous women with spontaneous labour, and found the important predictors for caesarean delivery to be high maternal age, high gestational age, low maternal height, high maternal weight (pre-pregnancy and at delivery), sonographic parameters, high estimated fetal weight, and fetal male sex [19]. Since prediction models often report an association between pre-pregnancy BMI and mode of delivery [20], it is noteworthy that for the included population the pre-pregnancy BMI has minor, but still important, impact on the risk for caesarean delivery. This finding is however consistent with the two studies described above [18, 19]. One could speculate if pre-pregnancy BMI is associated with the ability to go into active labour and therefore only have a minor impact on mode of delivery in the population of this study.

A perspective of this algorithm is that it is implementable at labour wards using electronic medical records. Pregnancy and delivery data, necessary for the algorithm, are routinely registered before and during labour. Due to the possible impact on the mode of delivery, it may be beneficial even though the decision regarding continuation or discontinuation of oxytocin only benefits 10% of the women. In 90% of the included women, the difference between oxytocin continuation and discontinuation in relation to the risk of caesarean delivery was less than 5% points. In these cases, shared decision making between the woman and the birth attendant would be necessary to decide on whether or not to discontinue oxytocin. In these women, the attitudes, the expectations, the women’s experience of pregnancy and labour, possible positive or negative attitudes in the delivery room, beliefs among caretakers, and the beliefs of the women as to whether vaginal birth is feasible should be included in shared decision making. A similar approach could be used if the risk of caesarean delivery is high, e.g., above 40%. It is unknown whether providing real-time AI assessments of likely outcome would change clinical practice. Some women might prefer to continue labour even with a small chance of vaginal delivery if she is in favor of minimizing intervention. In order to test the hypothesis that real-time AI risk assessments will change management it is first necessary to generate suitable and validated models that can predict risk, and we have presented one such model for future evaluation.

## Conclusion

In women reaching the active phase of induced labour during oxytocin stimulation, the AI-based algorithm identified women with both high and low risk of caesarean delivery. Furthermore, the algorithm identified 7.6% of the women who may have benefitted from oxytocin continuation and 2.5% who may have benefitted from oxytocin discontinuation. These results should be revaluated in further studies before implementation as a tool for an individually tailored clinical practice.

## Data Availability

Data may be available from the corresponding author in a reasonable request. However, owing to Danish legislation, data will be available only after approval by the Danish Data Protection Agency and with a signed access agreement.

## Abbreviations

AI: Artificial intelligence
AUROC: Area Under the Receiver Operating Characteristic
AUPRC: Area Under the Precision-Recall Curve
BMI: Body Mass Index
CD: Caesarean delivery
CI: Confidence interval
CONDISOX: Continued versus discontinued oxytocin stimulation in the active phase of labour
GCP: Good Clinical Practice
GPI: global parameter importance
PROM: Prelabour Rupture of membranes
SHAP: Shapley additive explanation
XGBoost: Extreme gradient boosting

## References

[1] Oláh KS, Steer PJ. The use and abuse of oxytocin. Obstet Gynaecol. 2015;4265–71. 10.1111/tog.12222.

[2] Boie S, Glavind J, Uldbjerg N, Steer PJ, Bor P. Continued versus discontinued oxytocin stimulation in the active phase of labour (CONDISOX): double blind randomised controlled trial. BMJ. Published online 14 April 2021:n716. 10.1136/bmj.n716. 10.1136/bmj.n716 PMC8044921 33853878

[3] Boie S, Glavind J, Velu AV (2018). Discontinuation of intravenous oxytocin in the active phase of induced labour. Cochrane Database Syst Rev.

[4] Dhombres F, Bonnard J, Bailly K, Maurice P, Papageorghiou AT, Jouannic JM. Contributions of Artificial Intelligence reported in obstetrics and gynecology journals: systematic review. J Med Internet Res. 2022;24(4). 10.2196/35465. 10.2196/35465 PMC9069308 35297766

[5] Levine LD, Downes KL, Parry S, Elovitz MA, Sammel MD, Srinivas SK (2018). A validated calculator to estimate risk of cesarean after an induction of labor with an unfavorable cervix. Am J Obstet Gynecol.

[6] Kamel RA, Negm SM, Youssef A (2021). Predicting cesarean delivery for failure to progress as an outcome of labor induction in term singleton pregnancy. Am J Obstet Gynecol.

[7] Danilack VA, Hutcheon JA, Triche EW (2020). Development and validation of a risk prediction model for cesarean delivery after labor induction. J Womens Health (Larchmt).

[8] de Carvalho LSF, Gioppato S, Fernandez MD et al. Machine Learning Improves the Identification of Individuals With Higher Morbidity and Avoidable Health Costs After Acute Coronary Syndromes. *Value Heal*. Published online 2020. 10.1016/j.jval.2020.08.2091. 10.1016/j.jval.2020.08.2091 33248512

[9] D’Ascenzo F, De Filippo O, Gallone G (2021). Machine learning-based prediction of adverse events following an acute coronary syndrome (PRAISE): a modelling study of pooled datasets. Lancet Published Online.

[10] Bzdok D, Altman N, Krzywinski M. Points of Significance: Statistics versus machine learning. *Nat Methods*. Published online. 2018. 10.1038/nmeth.4642. 10.1038/nmeth.4642 PMC6082636 30100822

[11] Boie S, Glavind J, Uldbjerg N (2019). CONDISOX-continued versus discontinued oxytocin stimulation of induced labour in a double-blind randomised controlled trial. BMC Pregnancy Childbirth.

[12] Chen T, Guestrin C. XGBoost: A scalable tree boosting system. In: *Proceedings of the ACM SIGKDD International Conference on Knowledge Discovery and Data Mining*.; 2016. 10.1145/2939672.2939785.

[13] Lundberg SM, Erion G, Chen H (2020). From local explanations to global understanding with explainable AI for trees. Nat Mach Intell Published Online.

[14] Ozenne B, Subtil F, Maucort-Boulch D (2015). The precision-recall curve overcame the optimism of the receiver operating characteristic curve in rare diseases. J Clin Epidemiol.

[15] Lundberg SM, Lee SI (2017). A unified approach to interpreting model predictions. Adv Neural Inf Process Syst.

[16] Lauritsen SM, Thiesson B, Jørgensen MJ (2021). The Framing of machine learning risk prediction models illustrated by evaluation of sepsis in general wards. Npj Digit Med.

[17] Steer PJ, Glavind J, Uldbjerg N, Bor P, Boie S (2023). Continued versus discontinued oxytocin stimulation in the active phase of induced labour: factors associated with unexpectedly high rates of conversion to open label oxytocin in the CONDISOX trial. BJOG Published Online January.

[18] Meyer R, Hendin N, Zamir M (2020). Implementation of machine learning models for the prediction of vaginal birth after cesarean delivery. J Matern Neonatal Med.

[19] Wie JH, Lee SJ, Choi SK (2022). Prediction of emergency Cesarean Section using machine learning methods: Development and External Validation of a nationwide Multicenter dataset in Republic of Korea. Life.

[20] Maršál K, Persson PH, Larsen T, Lilja H, Selbing A, Sultan B (1996). Intrauterine growth curves based on ultrasonically estimated foetal weights. Acta Paediatr Int J Paediatr.

